# Limited Thyroidectomy Achieves Equivalent Survival to Total Thyroidectomy for Early Localized Medullary Thyroid Cancer

**DOI:** 10.3390/cancers16234062

**Published:** 2024-12-04

**Authors:** Jessan A. Jishu, Mohammad H. Hussein, Salman Sadakkadulla, Solomon Baah, Yaser Y. Bashumeel, Eman Toraih, Emad Kandil

**Affiliations:** 1 School of Medicine, Tulane University, New Orleans, LA 70112, USA; jjishu@tulane.edu (J.A.J.); ssadakkadulla@tulane.edu (S.S.); sbaah@tulane.edu (S.B.); 2 Division of Endocrine and Oncologic Surgery, Department of Surgery, School of Medicine, Tulane University, New Orleans, LA 70112, USAekandil@tulane.edu (E.K.); 3 Ochsner Clinic Foundation, New Orleans, LA 70112, USA

**Keywords:** MTC, medullary thyroid cancer, thyroidectomy, lobectomy, T1N0M0, T1N1M0, SEER, endocrine

## Abstract

Background: The optimal surgical approach for localized T1 medullary thyroid cancer remains unclear. Total thyroidectomy is standard, but lobectomy and subtotal thyroidectomy may minimize mortality while maintaining oncologic control. Methods: This retrospective analysis utilized the National Cancer Institute’s Surveillance, Epidemiology, and End Results registry to identify 2702 MTC patients including 398 patients with T1N0/1M0 MTC treated with total thyroidectomy or lobectomy/subtotal thyroidectomy from 2000 to 2019. Cox regression analyses assessed thyroid cancer-specific and overall mortality. Results: The majority (89.7%) underwent total thyroidectomy, while 10.3% had lobectomy/subtotal thyroidectomy. Nodal metastases were present in 29.6%. Over a median follow-up of 8.75 years, no significant difference was observed in cancer-specific mortality (5.7% vs. 8.1%, *p* = 0.47) or overall mortality (13.2% vs. 12.8%, *p* = 0.95). On multivariate analysis, undergoing cancer-directed surgery was associated with significantly improved overall survival (HR 0.18, *p* < 0.001) and cancer-specific survival (HR 0.17, *p* < 0.001) compared to no surgery. However, no significant survival difference was seen between total thyroidectomy and lobectomy/subtotal thyroidectomy for overall mortality (HR 0.77, *p* = 0.60) or cancer-specific mortality (HR 0.44, *p* = 0.23). The extent of surgery also did not impact outcomes within subgroups stratified by age, gender, T stage, or nodal status. Delayed surgery >1 month after diagnosis was associated with worse overall survival (*p* = 0.012). Conclusions: For localized T1 MTC, lobectomy/subtotal thyroidectomy appears to achieve comparable long-term survival to total thyroidectomy in this population-based analysis. The selective use of limited thyroidectomy may be reasonable for low-risk T1N0/1M0 MTC patients. Delayed surgery is associated with worse survival and additional neck dissection showed no benefit for this select group of patients.

## Simple Summary

Medullary thyroid cancer is a rare but severe variant of thyroid cancer. The mainstay treatment for this condition is surgery to remove the entire thyroid gland, or total thyroidectomy. However, recent studies have found that a less extensive thyroidectomy may optimize survival outcomes for early localized tumors. We compared the survival and mortality outcomes between patients with such tumors who underwent total thyroidectomy and lobectomy/subtotal thyroidectomy to understand which surgical approach can improve survival rates in early-stage medullary thyroid cancer.

## 1. Introduction

Medullary thyroid cancer (MTC) is a neuroendocrine tumor originating from the parafollicular cells of the thyroid, accounting for approximately 5–10% of all thyroid cancers [1,2], and causing up to 14% of all thyroid disease-related deaths [3]. The hallmark of MTC is the secretion of calcitonin, which serves as a sensitive tumor marker for diagnosis and monitoring [4]. MTC can occur sporadically or be inherited in an autosomal dominant pattern as part of multiple endocrine neoplasia type 2 (MEN2) syndromes [2]. The hereditary forms are caused by activating mutations in the rearranged during transfection (*RET*) proto-oncogene [5].

Compared to well-differentiated thyroid cancers, MTC is more clinically aggressive with a high propensity for lymph node and distant metastases [6]. Regional lymph node involvement is present in up to 75% of patients at diagnosis and 10–15% have distant metastases to sites like liver, lung, and bone [7]. Five-year survival rates can reach up to 91% but decline considerably with extrathyroidal extension or advanced stage [8,9]. Given its clinical aggressiveness, complete surgical resection is the only potentially curative option [10,11,12].

Total thyroidectomy with central compartment neck dissection has traditionally been the standard approach as it ensures the clearance of bilateral disease [13]. However, total thyroidectomy has risks including permanent hypoparathyroidism, recurrent laryngeal nerve injury, and thromboembolisms, with incidence rates being higher in patients of older age or with more comorbidities [14]. Given the potential complications, interest has grown in lesser procedures such as lobectomy/subtotal thyroidectomy for small early tumors [15]. Indeed, these surgeries are less extensive and thus remove less tissue and have lower risks. However, there are concerns about locoregional recurrence and mortality when compared to total thyroidectomy [11].

The 2015 American Thyroid Association guidelines recommend total thyroidectomy for most MTCs to ensure adequate disease clearance [3]. However, optimal surgical management for early localized lesions is less defined, representing a knowledge gap. Although total thyroidectomy is standard, lobectomy/subtotal thyroidectomy may allow comparable oncologic control for T1 tumors while avoiding surgical complications [16]. This study aims to address this clinical equipoise by utilizing a national cohort to compare survival outcomes between total thyroidectomy and lobectomy/subtotal thyroidectomy specifically for localized T1N0/1M0 MTC.

## 2. Materials and Methods

### 2.1. Data Source

This retrospective cohort study utilized data from the Surveillance, Epidemiology, and End Results (SEER) program registry of the National Cancer Institute. SEER collects cancer incidence, treatment, and survival information from 17 population-based cancer registries, covering approximately 26.5% of the United States population (https://seer.cancer.gov/registries/data.html (accessed on 29 September 2023).

#### 2.1.1. Study Population

The study included patients who were diagnosed with pathologically confirmed MTC between 2000 and 2019. International Classification of Diseases for Oncology, 3rd edition (ICD-O-3) histology codes were used to identify MTC cases (codes 8345/3, 8346/3, 8347/3, 8510/3). Patients were included if they had histological T1 tumors with a maximum tumor dimension less than or equal to 2 cm. Patients were excluded if the MTC diagnosis was made only on autopsy or death certificate, had incomplete staging information, distant metastasis at diagnosis, prior or synchronous secondary malignancies, died within one month of diagnosis, or had unknown/missing data on survival.

#### 2.1.2. Study Variables

Baseline demographic (age, sex, race, ethnicity), socioeconomic (median household income, marital status, and geographical residency), tumor characteristics (year of diagnosis, histology, TNM stage, tumor size, extension, lymph node involvement, metastasis), and treatment details (type of surgery—total thyroidectomy, lobectomy/subtotal thyroidectomy, external beam radiation, systemic therapy) were extracted. Time to initiation of treatment dichotomized before and after 1 month from diagnosis was assessed.

#### 2.1.3. Study Outcomes

Patients undergoing total thyroidectomy were compared to those having lobectomy/subtotal thyroidectomy. The primary outcomes were overall mortality and thyroid cancer-specific mortality. Thyroid cancer-specific mortality was defined as death attributable to MTC documented in the SEER cause of death field. Overall mortality was defined as death from any cause. Secondary outcomes were recurrence and incidence of second primary malignancies.

#### 2.1.4. Statistical Analysis

Baseline characteristics were summarized using descriptive statistics. Thyroid cancer-specific mortality was estimated using Kaplan–Meier methods and differences between surgical groups (total thyroidectomy vs. lobectomy/subtotal thyroidectomy) were assessed using the log-rank test. Multivariable Cox proportional hazards regression was performed to determine the adjusted hazards ratio (HR) and 95% confidence interval (CI) for cancer-specific and overall mortality associated with type of surgery. Models were adjusted for potential confounders including age, sex, race, income, TNM stage, histology, systemic therapy, radiation, and treatment latency. Subgroup analysis by T and N stage was performed. The impact of various treatment approaches and latency time from diagnosis to treatment on survival were investigated. Two-sided *p* < 0.05 was considered statistically significant. All analyses were conducted using SPSS v28.0.

## 3. Results

### 3.1. Overall Cohorts

#### 3.1.1. Characteristics of the Study Population

A total of 398 patients with T1 MTC were identified, among whom 357 (89.7%) underwent surgery and 41 (10.3%) did not receive surgical treatment. The majority of patients were female (65.3%) and of White race (85.7%). The median age was 51 years (range 18–93 years), with 58.8% younger than 55 years. Pediatric patients accounted for 7.3% of the cohort (n = 29). The most common histological subtype was medullary carcinoma with amyloid stroma (52.5%), followed by medullary carcinoma without amyloid (41.2%). Over half had T1a tumors ≤1 cm (55.5%), while 44.5% were T1b >1–2 cm. Most were node-negative (N0, 70.4%), while 29.6% had nodal infiltration. Among the 398 patients, 357 (89.7%) received treatment while 41 (10.3%) did not undergo any intervention. Compared to the surgery group, those without surgery were older (mean age 61 vs. 50 years, *p* = 0.008), more likely male (53.7% vs. 32.5%, *p* = 0.009), urban residents (100% vs. 88.2%, *p* = 0.014), and presented with smaller T1a tumors (80.5% vs. 52.7%, *p* = 0.001) and less nodal involvement (N0 85.4% vs. 68.6%, *p* = 0.029). Details are presented in Table 1.

#### 3.1.2. Management Approach

The most common surgery was total thyroidectomy (n = 304, 85.2%), followed by lobectomy (n = 35, 9.8%) and subtotal thyroidectomy (n = 4, 1.1%). Regional lymph node dissection was performed in 46 patients (12.9%). Postoperative radiation was administered in 26 patients (7.3%), while 112 (31.4%) received systemic therapy.

#### 3.1.3. Risk of Recurrence

Only one case of recurrence was identified (Table 1), occurring in a 28-year-old Hispanic female with T1bN0M0 MTC treated initially with total thyroidectomy and neck dissection. The tumor recurred after 223 months.

#### 3.1.4. Risk of Second Primary Malignancies

**Figure 1:**
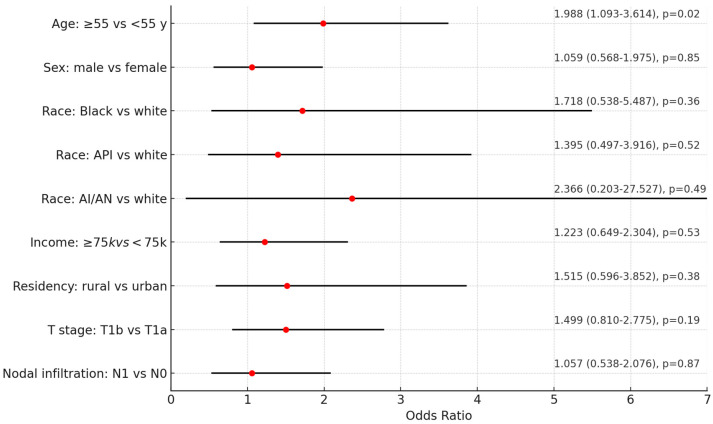
Independent risk factors for second primary malignancies. The forest plot visually represents the analysis of various independent risk factors for second primary malignancies. Logistic regression analysis was used. Odds ratio (OR) and 95% confidence interval (CI) are rported. Each line on the plot corresponds to a different risk factor, with the red dot indicating the odds ratio and the horizontal line representing the confidence interval (spanning from the lower to the upper limit). API: Asian or Pacific Islander, AI/AN: Am. Indian/Alaska Native.

Second primary malignancies occurred in 54 patients (13.6%), including 14 (3.5%) with subsequent thyroid cancers. Older age ≥55 years was associated with a 2-fold increased risk of second primaries (OR = 1.98, 95%CI = 1.09–3.61, *p* = 0.024), Figure 1.

#### 3.1.5. Survival Analysis

**Figure 2:**
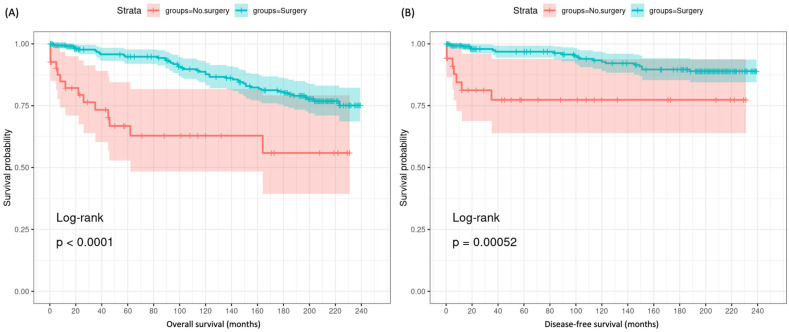
Kaplan–Meier survival curves in T1 MTC patients undergoing surgery vs. no surgery. (**A**) Overall survival. (**B**) Thyroid cancer-specific survival. Log-rank test was used.

Over a mean follow-up of 8.75 years, 60 patients died, including 27 (45%) from thyroid cancer. Those without surgery had significantly higher mortality, with 14 of 41 patients (34.1%) dying during follow-up compared to 46 of 357 patients (12.9%) in the surgery group (*p* = 0.001) (Table 1). Among the no surgery group, there was higher frequency of death due to diseases of the heart (7.3% vs. 1.1%), chronic obstructive pulmonary disease (2.4% vs. 0.6%), and other malignant cancers (2.4% vs. 0.0%). Kaplan–Meier analysis demonstrated more prolonged overall and thyroid cancer-specific survival times in the surgery cohort compared to no surgery in this T1 MTC cohort (both *p* < 0.001) (Figure 2). The surgery group had a median overall survival of 209.9 months (95% CI = 202.0–217.9 months) compared to 149.3 months (95% CI = 115.5–183.0 months) in the no surgery group, around 4.5 years longer. For thyroid cancer-specific survival, the surgery cohort had a median of 223.2 months (95% CI = 216.4–230.0 months) versus 181.1 months (95% CI = 148.4–213.8 months) for no surgery, over 3 years higher. Correlation analysis showed that tumor size did not significantly correlate with survival time (*r* = 0.056, *p* = 0.26). However, older age (*r* = −0.227, *p* < 0.001) and longer time from diagnosis to treatment (*r* = −0.242, *p* < 0.001) were associated with shorter survival.

On multivariable Cox regression analysis adjusting for demographic and tumor characteristics, undergoing cancer-directed surgery was strongly associated with improved survival outcomes in MTC patients. Specifically, surgery was linked to an 82% lower risk of overall mortality (adjusted HR = 0.18, 95%CI 0.09–0.35, *p* < 0.001) and an 83% lower risk of thyroid cancer-specific mortality (adjusted HR = 0.17, 95%CI 0.07–0.44, *p* < 0.001) compared to no surgery. Conversely, opting for surveillance corresponded to a 5.5 to 5.8 times higher risk of mortality, as shown in Table 2. Other factors associated with reduced mortality were younger age (<55 years), female sex, higher income (≥USD 75 k), and lack of lymph node infiltration (N0 stage). In contrast, older age (≥55 years) and presence of nodal metastases (N1 stage) were linked to significantly higher risks of both overall and cancer-specific mortality. T stage and rural versus urban residency showed no significant association.

### 3.2. Surgery Cohorts

#### 3.2.1. Comparison Between Total Thyroidectomy and Lobectomy/Subtotal Thyroidectomy

Table 3 shows that total thyroidectomy and less extensive surgery groups had comparable baseline demographic characteristics including age, sex, race, ethnicity, marital status, metropolitan status, and income. No significant differences were observed between surgical cohorts for any demographic factor examined (all *p* > 0.05).

Patients undergoing total thyroidectomy were more likely to have nodal disease at diagnosis compared to lobectomy/subtotal thyroidectomy (N1: 34.5% vs. 10.3%, *p* = 0.002). Total thyroidectomy patients were also more likely to receive systemic therapy (35.2% vs. 10.3%, *p* = 0.002) and experience delayed time to treatment over 1 month (23.3% vs. 5.3%, *p* = 0.010), Table 3. However, no significant differences were observed between surgical groups in terms of second primary malignancy rates (15.5% vs. 7.7%, *p* = 0.24), recurrence (0.3% vs. 0%, *p* = 0.72), overall mortality (13.2% vs. 12.8%, *p* = 0.95), or thyroid cancer-specific mortality (5.7% vs. 8.1%, *p* = 0.47).

#### 3.2.2. Impact of Type of Cancer Surgery on Survival

**Figure 3:**
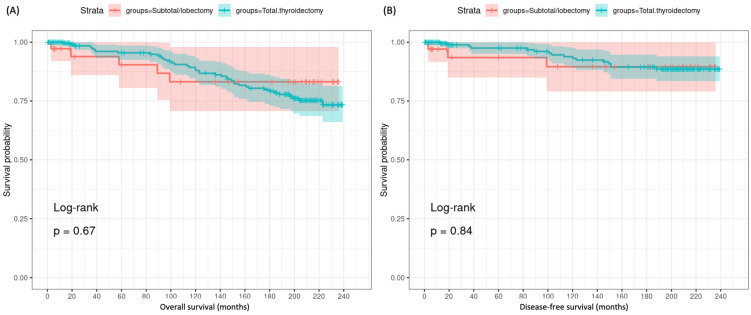
Kaplan–Meier survival curves in T1 MTC patients undergoing total thyroidectomy versus lobectomy/subtotal thyroidectomy. (**A**) Overall survival. (**B**) Thyroid cancer-specific survival. Log-rank test was used.

As depicted in Kaplan–Meier analysis, the extent of surgery did not impact long-term mortality outcomes in the T1 MTC cohort (Figure 3). For overall survival, there was no significant difference between lobectomy/subtotal thyroidectomy and total thyroidectomy (205.8 months, 95%CI: 181.3–230.3 versus 209.6 months, 95%CI: 200.8–218.3, *p* = 0.67). Similarly, for thyroid cancer-specific survival, no significant difference was observed between surgical groups (216 months, 95%CI: 194.3–237.8 versus 223.7 months, 95%CI: 216.3–231.1, *p* = 0.84). On multivariable Cox regression analysis adjusting for demographic and tumor factors, there was no significant difference in overall mortality (adjusted HR = 0.77, 95%CI 0.29–2.03, *p* = 0.60) and thyroid cancer-specific mortality (adjusted HR = 0.44, 95%CI 0.11–1.68, *p* = 0.23) between total thyroidectomy and lobectomy/subtotal thyroidectomy groups (Table 4). This indicates that after accounting for confounding factors like age, gender, income, etc., the extent of surgery did not independently impact mortality outcomes. In contrast, nodal disease and older age were independent poor prognostic factors for MTC. The presence of nodal infiltration (N1 vs. N0) was associated with a significantly higher risk of overall mortality (HR = 3.80, 95% CI 2.03–7.10, *p* < 0.001) and thyroid cancer-specific mortality (HR = 7.76, 95% CI 2.66–22.61, *p* < 0.001). Older age (≥ 55 vs. <55 years) also increased the risk of overall (HR = 3.03, 95% CI 1.66–5.52, *p* < 0.001) and cancer-specific mortality (HR = 2.63, 95% CI 1.04–6.68, *p* = 0.042). Other factors like income, gender, T stage, and residency were not significantly associated with mortality in the multivariable model.

#### 3.2.3. Impact of Concomitant Neck Dissection on Survival

We performed subgroup analyses stratified by whether patients underwent concomitant regional lymph node dissection along with primary surgery or isolated cancer-directed surgery without neck dissection. In the subgroup undergoing isolated primary surgery without neck dissection, there was no significant difference in overall survival between total thyroidectomy versus lobectomy/subtotal thyroidectomy (HR 1.611, 95% CI 0.572–4.543, *p* = 0.37). Similarly, in the subgroup undergoing primary surgery with concomitant neck dissection, overall survival did not differ significantly between total thyroidectomy and limited surgery (HR 0.352, 95% CI 0.042–2.931, *p* = 0.33). Therefore, concomitant neck dissection did not modify the relationship between type of primary thyroid surgery and survival outcomes in T1 MTC patients. Total thyroidectomy did not confer a survival advantage over lobectomy/subtotal thyroidectomy regardless of whether or not patients underwent additional lymph node dissection.

#### 3.2.4. Impact of Adjuvant Radiotherapy, Radioactive Iodine Ablation, and Systemic Therapy on Survival

Beyond the extent of initial surgery, the use of adjuvant therapies after surgery may potentially influence survival outcomes. Therefore, we analyzed the comparative impact of external beam radiation, radioactive iodine (RAI) ablation, and systemic therapy on mortality (Table 5). On univariate analysis, external beam radiation was associated with significantly higher overall mortality (HR = 4.42, 95%CI 1.57–12.48, *p* = 0.005) and thyroid cancer-specific mortality (HR = 3.45, 95%CI 1.02–14.64, *p* = 0.049) compared to no radiation. The mean overall (mean 144.2 vs. 204.7 months, *p* = 0.03) and cancer-specific (mean 163 vs. 220.6, *p* = 0.047 months) survival times were shorter for patients receiving external beam radiation versus no radiation (Table 6). However, after adjusting for confounders in a multivariate model, radiation therapy was no longer significantly associated with overall mortality (adjusted HR = 1.51, 95%CI = 0.48–4.81, *p* = 0.48) or cancer-specific mortality (adjusted HR = 3.21, 95%CI = 0.58–17.88, *p* = 0.18), indicating that radiation itself did not independently impact survival. Neither RAI ablation nor systemic therapy were significantly associated with mortality outcomes on univariate or multivariate analysis. The mean survival times did not differ significantly between patients receiving RAI versus no RAI. For systemic therapy, estimates were unstable due to few events, but mean survival times were numerically shorter with systemic therapy versus no systemic therapy (Table 5 and Table 6).

#### 3.2.5. Impact of Type of Cancer Surgery on Survival Stratified by Patient and Tumor Factors

**Figure 4:**
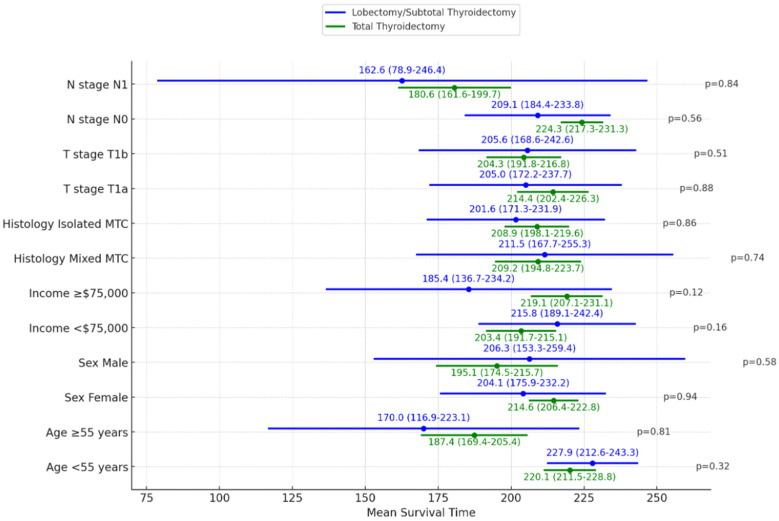
Subgroup analysis comparing overall survival times in total thyroidectomy and lobectomy/subtotal thyroidectomy T1 MTC patients. The values of the mean in months and confidence intervals (CIs) are displayed on the plot for both lobectomy/subtotal thyroidectomy (blue) and total thyroidectomy (green) treatments. Log-rank test was used for comparisons.

We performed subgroup analyses comparing overall survival times between total thyroidectomy and lobectomy/subtotal thyroidectomy after stratifying patients according to age, sex, income, histology, T stage, and nodal status (Figure 4). The extent of surgery did not impact overall survival within any of the subgroups examined. Specifically, in patients <55 years, the mean overall survival did not differ significantly between lobectomy and total thyroidectomy (227.9 vs. 220.1 months, *p* = 0.32). Similarly, for those ≥55 years, survival was comparable between lobectomy and total thyroidectomy (170.0 vs. 187.4 months, *p* = 0.81). Among females, limited surgery and total thyroidectomy had similar survival times (204.1 vs. 214.6 months, *p* = 0.94). For males, survival was also equivalent between lobectomy and total thyroidectomy (206.3 vs. 195.1 months, *p* = 0.58). In lower-income patients, the mean survival did not differ significantly between lobectomy and total thyroidectomy (215.8 vs. 203.4 months, *p* = 0.16). The same was true for higher-income patients (185.4 vs. 219.1 months, *p* = 0.12). Additionally, no significant differences in overall survival were observed after stratifying by histological subtype, T stage, or N stage (all *p* > 0.05). The data demonstrate that total thyroidectomy did not confer a survival advantage compared to lobectomy/subtotal thyroidectomy in T1 MTC patients with any demographic or tumor characteristic.

#### 3.2.6. Impact of Surgical Timing on Survival

**Figure 5:**
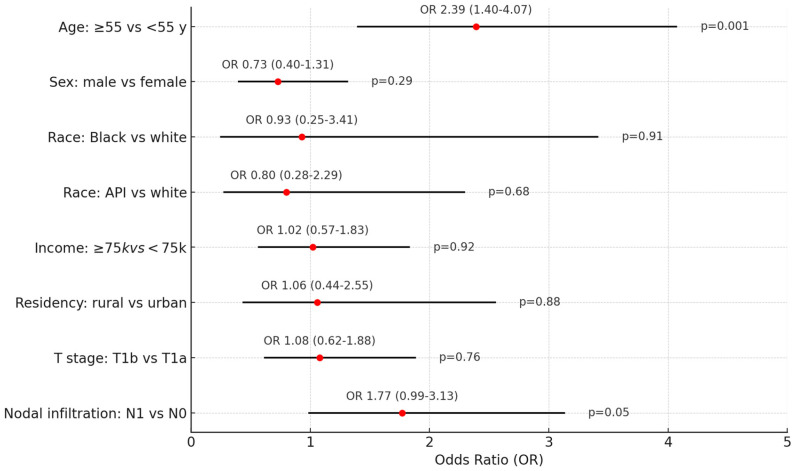
Predictors of delayed onset of treatment in MTC patients. API: Asian or Pacific Islander. Logistic regression analysis was performed and odds ratio (OR) and 95% confidence intervals (CIs) were reported.

Beyond examining the extent of surgery, we also analyzed the impact of timing of initial treatment on survival outcomes in T1 MTC patients. Among the 357 patients who underwent surgery, 278 (77.9%) were treated within 1 month of diagnosis (early treatment), while 74 (20.7%) had delayed treatment exceeding 1 month. Patients with delayed treatment were more likely to be older (55.4% versus 35.6%, *p* = 0.003), present with nodal disease (41.9% versus 29.5%, *p* = 0.05), and receive systemic therapy (51.4% versus 26.3%, *p* < 0.001), as seen in Table 7. Older age (OR = 2.39, 95%CI = 1.40–4.07, *p* = 0.001) and nodal positivity (OR = 1.77, 95%CI = 0.99–3.13, *p* = 0.05) were independent risk factors for delayed treatment exceeding 1 month on multivariate analysis (Figure 5).

**Figure 6:**
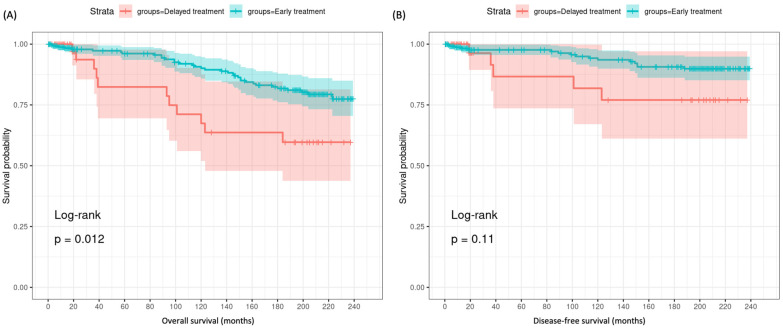
Kaplan–Meier survival curves in T1 MTC patients according to the timing of treatment. (**A**) Overall survival. (**B**) Thyroid cancer-specific survival. Log-rank test was used.

The rates of second malignancy (12.2% vs. 15.1%, *p* = 0.58) and recurrence (0% vs. 0.4%, *p* = 0.61) did not differ significantly based on treatment timing. However, Kaplan–Meier analysis demonstrated a potential survival advantage for earlier surgical treatment within 1 month versus delayed surgery after 1 month. Timely surgery within 1 month of diagnosis was associated with improved overall survival compared to delayed surgery over 1 month (213.9 vs. 174.2, *p* = 0.012). A similar favorable trend was observed for cancer-specific survival with early versus late surgery, although this did not reach statistical significance (224.9 vs. 197.6, *p* = 0.11), Figure 6.

### 3.3. Surgery Cohorts with T1N0 Stage

#### 3.3.1. Patient Characteristics

Next, we explored the impact of extent of surgery on MTC patients without regional lymph node metastasis. In this N0 subgroup, the majority of node-negative patients had T1a tumors (65%) compared to T1b (35%). Recurrence was rare, seen in just one patient (0.4%). Rates of subsequent cancers were low (13.2%). Subsequent thyroid cancers of different histology occurred in only 3.9% of T1N0 patients. The vast majority were alive at the last follow-up. Overall mortality was 11.1% (N = 31), and only 3.9% (N = 11) had thyroid cancer-specific mortality. Another leading cause of death was heart disease.

#### 3.3.2. Survival Analysis

Table 8 compares clinicopathologic factors between survived (N = 249) and deceased (N = 31) T1N0 MTC patients. Older age (≥ 55 years) was significantly associated with mortality, with 67.7% of deaths occurring in patients ≥ 55 years compared to 38.6% of survived patients (*p* = 0.003). Additionally, deceased patients were less likely to undergo cancer-directed surgery, with only 64.5% receiving surgery versus 90.4% of survived patients (*p* < 0.001). An interesting finding was that radiation therapy was more frequently used in deceased patients, with 16.1% receiving radiation compared to just 4.0% of survived patients (*p* = 0.016). Meanwhile, systemic therapy was significantly less common in deceased patients, with only 3.2% receiving it versus 26.9% of survived patients (*p* = 0.002). However, no differences were observed between deceased and survived groups for recurrence or second primaries. In summary, older age and lack of definitive surgery appear to increase mortality risk in node-negative T1N0 MTC based on these preliminary data.

#### 3.3.3. Comparison of Total Thyroidectomy Versus Limited Surgery in T1N0 MTC

We performed subgroup analyses specifically evaluating the impact of total thyroidectomy versus lobectomy/subtotal thyroidectomy on survival outcomes in T1N0 MTC patients without lymph node metastases. Multivariable Cox regression was used to assess overall and cancer-specific mortality, adjusting for demographic and clinicopathologic factors. Extent of surgery was not significantly associated with overall survival (adjusted HR 0.805, 95% CI 0.223–2.898, *p* = 0.74) or cancer-specific survival (adjusted HR 0.222, 95% CI 0.036–1.359, *p* = 0.103) when comparing total thyroidectomy to lobectomy/subtotal thyroidectomy. These findings indicate that more extensive surgery does not confer improved mortality over limited thyroidectomy for T1N0 disease after accounting for confounders. The only factor independently associated with worse overall survival was older age (adjusted HR 1.073, 95%CI 1.03–1.118, *p* < 0.001). Other characteristics like gender, income, residency, and T stage did not significantly impact outcomes, as shown in Table 9.

## 4. Discussion

The optimal initial surgical management of small, localized T1 medullary thyroid cancers remains highly controversial. The 2015 American Thyroid Association guidelines provide no definitive recommendations regarding the ideal approach for these early tumors, representing an important knowledge gap [3,17,18]. Total thyroidectomy has traditionally been the standard approach, but interest has grown in lesser procedures like lobectomy for selected patients to optimize survival. Our large, contemporary population-based analysis helps provide more definitive real-world data regarding this topic.

In our SEER cohort of over 350 T1N0/1M0 MTC patients, we did not identify significant differences in long-term overall survival or thyroid cancer-specific mortality between total thyroidectomy and lobectomy/subtotal thyroidectomy groups for treating early-stage MTC. Overall survival was equivalent at 86.8% after total thyroidectomy compared to 87.2% for lobectomy (*p* = 0.95). The cancer-specific mortality rates were also similar at 5.7% for total thyroidectomy versus 8.1% for lesser surgery (*p* = 0.47). These findings support the oncologic adequacy of lobectomy for low-risk T1 MTC when performed in appropriately selected patients. Previous studies corroborate this by finding that lobectomy was an effective surgical treatment for low-risk MTC in both sporadic and hereditary cases [19,20].

These findings have echoed conclusions made from recent studies based in China. Liang et al. identified patients treated for early localized MTC tumors less than 4 cm without any nodal or distant metastasis from the SEER database and validation cohort from three institutions in China, finding no significant associations between the type of surgery and long-term survival [16]. Yang et al. found that older patients (≥ 60 years) did not receive additional survival benefits from undergoing total thyroidectomy whereas younger patients (<60 years) did experience improved survival, suggesting that the age factor contributed more to overall survival when compared to the type of surgery above this threshold [21]. We found the statistical age threshold to reconstruct age from a quantitative to categorical variable to be 55 years instead of 60. More notably, however, our study found no additional survival benefit from total thyroidectomy between younger and older patients.

Overall, the present study focuses on overall and thyroid cancer-specific mortality as the primary outcomes. This intentional emphasis was inspired by a recent study which utilized the SEER database to confirm that the incidence of early-stage tumors, implementation rate of total thyroidectomy, and mortality trends of MTC in the United States have been consistently and concomitantly increasing since 2000 [22]. Although locoregional recurrence is a concern, recent studies have revealed this risk being higher for late-stage tumors compared to early-stage ones for both hereditary and sporadic MTC [23,24]. Indeed, this corroborates our study’s finding of only one patient exhibiting locoregional recurrence. Although we appreciate that there may be greater value in prioritizing survival when treating T1N0/1M0 MTC, especially in high-risk populations such as older patients or those delaying treatment as our study has also revealed, recurrence should still be addressed.

The notion of de-escalating surgery while maintaining oncologic efficiency has been increasingly favored in the recent literature. This is especially true for sporadic MTC, as highlighted by recent German studies which confirm the viability of a frozen section-guided lobectomy with ipsilateral central node dissection [25,26]. This novel technique helps clinicians confirm the absence of tumor desmoplasia and assist in their clinical decision-making to perform a less-than-total thyroidectomy while still achieving a biochemical cure. However, this technique is limited to desmoplasia-negative sporadic MTC in institutions equipped with pathology units capable of performing frozen section analysis. Performing such analysis at other institutions is encouraged to validate this practice, but this merely underscores the increasing demand and use of less extensive thyroid surgery to achieve sufficient oncological therapy.

Our study also demonstrates a survival benefit of surgical treatment compared to surveillance alone for T1 MTC, with over 5-fold higher mortality without resection. Older age and delays exceeding 1 month until surgery negatively impacted outcomes. Timely surgery, especially within 1 month of diagnosis, was associated with significantly improved survival compared to delays beyond 1 month, which was the statistical time threshold. This aligns with prior studies showing better outcomes, with early resection attributed to preventing locoregional spread during a critical curative window [17,27]. Notably, older patients (≥55 years) and those with nodal metastases were prone to treatment delays, representing high-risk groups where expeditious surgery may be most critical. We must acknowledge that there is a slight possibility of bias in this delayed timing of surgery within older patients due to recurrence remaining even after multivariate analysis. In other words, the possibility of independence between old age and delayed timing cannot entirely be ruled out due to the single case of recurrence that may have occurred due to old age. However, our conclusion corroborates our previous study investigating the influence of surgery refusal, which also demonstrated that older adults were more likely to refuse surgery, and that neither refusing or delaying surgery had a significant association with disease recurrence compared to early surgery in other thyroid cancer variants [28]. Thus, this makes the association between timely surgery and improved outcomes more genuine. Furthermore, Mathiesen et al. demonstrated that such delays can increase the number of metastasized lymph nodes and decrease the chance of disease-free survival, as shown by [29]. The reasons for delays in node-positive patients likely relate to more extensive preoperative staging, scheduling issues with complex surgery, and efforts to optimize higher risk patients. Shojaie et al. emphasize patient-centered reasoning, claiming that such delays can also be due to worry, financial concerns, and fear of disclosing the diagnosis with family members that delay treatment [30]. These observations not only support the importance of prompt surgery, be it either total thyroidectomy or lobectomy/subtotal thyroidectomy, but also accentuate the importance of education and encouragement in order to develop personalized treatment plans that provide the patients some comfort.

Beyond the extent of initial surgery, adjuvant treatments may also influence survival outcomes [17,31,32,33,34,35]. We analyzed the impact of additional radiation and systemic therapies after surgery in our cohort. On univariate analysis, external beam radiation was associated with higher mortality compared to no radiation. The reasons for this are unclear, but it could signify radiation being used for palliation or high-risk features not captured in these data. However, after adjusting for confounders, radiation therapy was not an independent predictor of mortality. As a result, beam radiation itself did not impact survival as the association was attenuated in multivariate models. RAI ablation also showed no significant association with mortality outcomes, which is unsurprising given that the parafollicular cells that give rise to MTC are unable to uptake RAI. Indeed, the 2015 American Thyroid Association guidelines explain that RAI ablation is not indicated following thyroidectomy in MTC [3]. In the current data, however, such a therapy may have been used in select cases where MTC was present with other thyroid cancer subvariants, such as papillary or follicular. There have been several reports of RAI ablation being used in mixed MTC cases where both medullary and some other subtype were present, although its use and efficacy in this setting have not been extensively investigated [36]. Unfortunately, the relative paucity of reported mixed MTC cases in our study and in general precluded a formal subanalysis for survival outcomes for this specific cohort. Sandilos et al. performed the largest series of mixed MTC cases to date and found that mixed medullary-papillary tumors demonstrated higher rates of 10-year overall survival than pure medullary tumors, while mixed medullary-follicular tumors demonstrated lower rates, although this study did not consider the influences of staging or adjuvant therapies such as RAI ablation due to the dearth of cases [37]. Although these findings suggest that the duplicity of cancer subvariants may affect prognosis, the role of adjuvant therapy in this association is uncertain. Systemic therapy did not emerge as a predictor of survival, although estimates were unstable due to few events in that subgroup. However, we did see that systemic therapy was significantly more common in survived than in deceased patients. The reduced systemic treatment rates could suggest undertreatment in some high-risk patients. However, this is uncertain given that there is no recommendation to guide the decision to perform systemic therapy in early-stage MTC. Additionally, in subgroup analyses by the type of surgery, we did not find a significant interaction between adjuvant therapy and surgical extent on thyroid cancer mortality. The survival difference between total thyroidectomy and lobectomy was consistent regardless of these therapies in our T1N0/1M0 MTC cohort. Larger studies are still needed to clarify if adjuvant treatments have differential effects depending on the extent of initial surgery of these early localized tumors.

Unfortunately, as our study utilized the SEER database, which lacks preoperative and postoperative procalcitonin levels, we were unable to accurately assess biochemical cure or recurrence. Indeed, procalcitonin levels have been shown to be reliable indicators for achieving biochemical cure or recurrence. However, a few studies argue that some discrepancies exist, such as analytical sensitivities differing between assay kits or difficulty establishing standardized cutoff values [4,38]. Using procalcitonin to correctly identify MTC cases may be difficult, especially at an early stage or small size. Censi et al. noted that procalcitonin levels were below the threshold value in six patients within their cohort of 43 MTC patients, with all but one of these patients being T1N0/1M0 [38]. To our knowledge, this is the only documented study in the literature with information comparing tumor staging with procalcitonin. Jeong et al. recently found that among 23 cases of MTC, only 1 case with a tumor size of 0.3 cm revealed a preoperative procalcitonin below the cutoff, while the correlation coefficient between the size of the remaining MTC tumors and preoperative procalcitonin was only 0.599 [39]. As such, there is a possibility that procalcitonin levels may not necessarily be sensitive in patients at an early stage, small tumor size, or low burden of disease. This may also be due to the contention that early-stage or small tumors have not yet progressed to the extent where procalcitonin levels would truly be elevated. The 2015 American Thyroid Association guidelines currently have no recommendation regarding the use of procalcitonin in management, although they have labeled it as a topic of interest [3]. As such, further studies are needed to explain any association between procalcitonin and early localized MTC in order to accurately conclude if procalcitonin can reliably predict a true biochemical cure at an early stage.

The SEER database also lacks information regarding the family history of MTC or genetic factors, including the status of *RET* gene mutations. It is well known that mutations in the *RET* proto-oncogene result in all familial cases and up to 50% of sporadic MTC [40]. To date, this gene serves as the only reliable genetic prognostic factor, as certain mutations, the most common one being localized on codon 918, are more aggressive and lead to worse survival outcomes [41]. Interestingly, recent studies have revealed how *RET* mutation levels were shown to be significantly low in small-size tumors when compared to large-size tumors [42,43]. Romei et al. discovered in two separate studies that the prevalence of the M918T *RET* mutation was much lower in tumors smaller than 2 cm than in larger tumors with no significant difference when the smaller tumors were subdivided into tumors smaller than 1 cm and 1–2 cm, suggesting that the mutation frequently occurs in the late stages of tumor proliferation and transformation [44,45]. Such findings suggest that the prevalence of such mutations in our study’s cohort may just as well be low. However, more studies are required to investigate the influence of *RET* mutations on the long-term outcomes of early localized MTC.

The potential advantages of our study include the large sample size exceeding 350 T1N0/1M0 MTC patients, providing substantial statistical power. The median follow-up of over 7 years allows assessment of long-term oncologic outcomes. The design of the SEER study, which is population-based and spans a wide geographic area over two decades, enhances the generalizability of our findings to everyday clinical practice. The study helps address a major knowledge gap regarding optimal initial surgery for localized T1 MTCs, with current guidelines lacking definitive recommendations. Unique strengths include the assessment of timing from diagnosis to treatment as well as the effects of adjuvant therapies by surgical type.

However, limitations include the retrospective design without full data on potential confounders. We lacked information on the etiology of MTC (sporadic or hereditary), surgical complications, postoperative calcitonin levels, and *RET* mutation status, which could allow more comprehensive analysis. Furthermore, although our overall sample size was very large, certain subgroups were relatively small. Larger prospective multicenter studies collecting granular molecular and clinicopathologic data could help overcome these limitations and allow personalized identification of patients likely to benefit from lobectomy/subtotal thyroidectomy versus total thyroidectomy for early localized MTC.

Our study has important clinical implications regarding the surgical management of early-stage MTC. The equivalent long-term survival achieved with lobectomy/subtotal thyroidectomy supports consideration of these lesser procedures for localized T1N0/1M0 tumors in appropriately chosen patients. Less extensive surgery may allow avoidance of morbidity from total thyroidectomy while maintaining oncologic efficacy based on our observations. Regardless, rigorous patient selection criteria accounting for factors like mutational status, biomarker levels, and comorbid conditions are necessary. A personalized approach weighing the potential risks and benefits of lobectomy versus total thyroidectomy is warranted. Patients should be cautiously counseled about the options with a thorough discussion of the current evidence.

## 5. Conclusions

Our large population-based SEER analysis did not demonstrate superior survival with total thyroidectomy compared to lobectomy/subtotal thyroidectomy for T1N0/1M0 MTC. Equivalent long-term cancer-specific and overall mortality were achieved with lesser surgery. These findings support the selective use of less extensive thyroid surgery in appropriately chosen low-risk patients to potentially minimize surgical morbidity. Additional large multicenter studies collecting detailed molecular and clinicopathologic data are warranted to confirm our results and allow comprehensive risk stratification. Overall, a personalized, risk-adapted approach accounting for both oncologic and quality of life factors is appropriate when recommending surgical options for T1 medullary tumors.

## Figures and Tables

**Table 1:** Clinicopathological characteristics and disease outcomes of T1 MTC patients.

Characteristics	Levels	Total Cohort (N = 398)	Surgery (N = 357)	No Surgery (N = 41)	*p*-Value
Demographics					
Age (years)	<55 years	234 (58.8)	218 (61.1)	16 (39.0)	0.008
	≥55 years	164 (41.2)	139 (38.9)	25 (61.0)	
Gender	Female	260 (65.3)	241 (67.5)	19 (46.3)	0.009
	Male	138 (34.7)	116 (32.5)	22 (53.7)	
Race	White	341 (85.7)	308 (86.3)	33 (80.5)	0.58
	Black	23 (5.8)	19 (5.3)	4 (9.8)	
	API	31 (7.8)	27 (7.6)	4 (9.8)	
	AI/AN	3 (0.8)	3 (0.8)	0 (0.0)	
Ethnicity	Not Hispanic/Latino	344 (86.4)	311 (87.1)	33 (80.5)	0.23
	Hispanic/Latino	54 (13.6)	46 (12.9)	8 (19.5)	
Marital status	Married	197 (60.1)	14 (45.2)	197 (60.1)	0.52
	Domestic partner	5 (1.5)	0 (0.0)	5 (1.5)	
	Separated	4 (1.2)	1 (3.2)	4 (1.2)	
	Divorced	19 (5.8)	3 (9.7)	19 (5.8)	
	Widowed	20 (6.1)	2 (6.5)	20 (6.1)	
	Single	83 (25.3)	11 (35.5)	83 (25.3)	
Residency	Urban	356 (89.4)	315 (88.2)	41 (100.0)	0.014
	Rural	42 (10.6)	42 (11.8)	0 (0.0)	
Household annual income	USD 75,000+	123 (34.5)	19 (46.3)	123 (34.5)	0.60
	USD 70,000–74,999	41 (11.5)	2 (4.9)	41 (11.5)	
	USD 65,000–69,999	43 (12.0)	6 (14.6)	43 (12.0)	
	USD 60,000–64,999	60 (16.8)	5 (12.2)	60 (16.8)	
	USD 55,000–59,999	24 (6.7)	4 (9.8)	24 (6.7)	
	USD 50,000–54,999	24 (6.7)	1 (2.4)	24 (6.7)	
	USD 45,000–49,999	21 (5.9)	2 (4.9)	21 (5.9)	
	USD 40,000–44,999	9 (2.5)	2 (4.9)	9 (2.5)	
	USD 35,000–39,999	6 (1.7)	0 (0.0)	6 (1.7)	
	<USD 35,000	6 (1.7)	0 (0.0)	6 (1.7)	
Pathological data					
Histological variant	8345/3: MTC with amyloid stroma	209 (52.5)	199 (55.7)	10 (24.4)	<0.001
	8346/3: Mixed MTC-FTC	9 (2.3)	9 (2.5)	0 (0.0)	
	8347/3: Mixed MTC-PTC	16 (4.0)	15 (4.2)	1 (2.4)	
	8510/3: MTC without amyloid stroma	164 (41.2)	134 (37.5)	30 (73.2)	
T staging	T1a	221 (55.5)	188 (52.7)	33 (80.5)	0.001
	T1b	177 (44.5)	169 (47.3)	8 (19.5)	
N staging	N0	280 (70.4)	245 (68.6)	35 (85.4)	0.029
	N1	118 (29.6)	112 (31.4)	6 (14.6)	
Disease outcomes					
Recurrence	Positive recurrence	1 (0.3)	1 (0.3)	0 (0.0)	0.73
Second primary malignancy	Any cancer	54 (13.6)	51 (14.3)	3 (7.3)	0.33
	Thyroid cancer	14 (3.5)	14 (3.9)	0 (0.0)	0.38
Survival status	Alive	338 (84.9)	311 (87.1)	27 (65.9)	0.001
	Died	60 (15.1)	46 (12.9)	14 (34.1)	
Cause of death	Thyroid cancer	27 (45.0)	20 (43.5)	7 (50.0)	0.67
	Other causes	33 (55.0)	26 (56.5)	7 (50.0)	

Data are presented as number and percentage. Two-sided Chi-Square test was used. Statistical significance was set at *p*-value < 0.05. API: Asian or Pacific Islander, AI/AN: Am. Indian/Alaska Native, MTC: medullary thyroid cancer, FTC: follicular thyroid cancer, PTC: papillary thyroid cancer.

**Table 2:** Impact of undergoing cancer-directed surgery on survival in MTC patients.

Risk Factor	Overall Mortality			Thyroid Cancer-Specific Mortality		
	HR	95%CI	*p*-Value	HR	95%CI	*p*-Value
Age: ≥55 vs. <55 y	3.85	(2.24–6.60)	<0.001	3.77	(1.70–8.37)	0.001
Sex: male vs. female	1.70	(1.01–2.87)	0.046	1.72	(0.79–3.76)	0.17
Income: ≥USD 75 k vs. <USD 75 k	0.46	(0.25–0.84)	0.012	0.38	(0.15–0.96)	0.041
Residency: rural vs. urban	1.21	(0.53–2.75)	0.66	1.56	(0.44–5.53)	0.49
T stage: T1b vs. T1a	1.13	(0.65–1.97)	0.66	0.77	(0.33–1.75)	0.53
Nodal infiltration: N1 vs. N0	3.35	(1.91–5.87)	<0.001	5.97	(2.48–14.33)	<0.001
Surgery vs. none	0.18	(0.09–0.35)	<0.001	0.17	(0.07–0.44)	<0.001

Cox regression analysis was performed and hazards ratio (HR) and 95% confidence interval (CI) were reported.

**Table 3:** Comparison between total thyroidectomy and less aggressive surgery.

Characteristics	Levels	Lobectomy and Subtotal (N = 39)	Total Thyroidectomy (N = 304)	*p*-Value
Demographics				
Age (years)	Pediatric	1 (2.6)	26 (8.6)	0.34
	Adults	38 (97.4)	278 (91.4)	
	<55 years	22 (56.4)	185 (60.9)	0.61
	≥55 years	17 (43.6)	119 (39.1)	
Gender	Female	29 (74.4)	203 (66.8)	0.37
	Male	10 (25.6)	101 (33.2)	
Race	White	33 (84.6)	261 (85.9)	0.48
	Black	1 (2.6)	18 (5.9)	
	API	4 (10.3)	23 (7.6)	
	AI/AN	1 (2.6)	2 (0.7)	
Ethnicity	Not Hispanic/Latino	37 (94.9)	265 (87.2)	0.19
	Hispanic/Latino	2 (5.1)	39 (12.8)	
Marital status	Married	24 (70.6)	169 (59.3)	0.15
	Domestic partner	0 (0.0)	5 (1.8)	
	Separated	0 (0.0)	4 (1.4)	
	Divorced	3 (8.8)	15 (5.3)	
	Widowed	4 (11.8)	16 (5.6)	
	Single	3 (8.8)	76 (26.7)	
Metropolitan	Metropolitan >1 M pop	19 (48.7)	172 (56.6)	0.18
	Metropolitan >250 K^−1^ M	9 (23.1)	81 (26.6)	
	Metropolitan of <250 K	2 (5.1)	21 (6.9)	
	Non-Metropolitan adj to Metropolitan	5 (12.8)	19 (6.3)	
	Non-Metropolitan not adj to Metropolitan	4 (10.3)	11 (3.6)	
Household annual income	<USD 75,000	27 (69.2)	197 (64.8)	0.72
	≥USD 75,000	12 (30.8)	107 (35.2)	
Pathological data				
Histological variant	MTC	36 (92.3)	283 (93.1)	0.74
	Mixed MTC	3 (7.7)	21 (6.9)	
T staging	T1a	25 (64.1)	152 (50.0)	0.13
	T1b	14 (35.9)	152 (50.0)	
N staging	N0	35 (89.7)	199 (65.5)	0.002
	N1	4 (10.3)	105 (34.5)	
Management				
Surgery at other sites	Regional sites	3 (7.7)	51 (16.8)	0.17
	Regional lymph nodes	3 (7.7)	43 (14.1)	0.33
	Distant sites	1 (2.6)	20 (6.6)	0.49
	Distant lymph nodes	1 (2.6)	17 (5.6)	0.71
Radiation therapy	Negative	36 (92.3)	283 (93.1)	0.61
	Beam radiation	2 (5.1)	8 (2.6)	
	Radioactive ablation	1 (2.6)	13 (4.3)	
Radiation therapy with surgery	Positive	3 (7.7)	20 (6.6)	0.74
Systemic therapy with surgery	Positive	4 (10.3)	107 (35.2)	0.002
Time to treatment	<1 month	36 (94.7)	231 (76.7)	0.010
	≥1 month	2 (5.3)	70 (23.3)	
Disease outcomes				
Second primary malignancy	Any cancer	3 (7.7)	47 (15.5)	0.24
	Thyroid cancer	1 (2.6)	13 (4.3)	0.61
Recurrence	Positive recurrence	0 (0.0)	1 (0.3)	0.72
Survival status	Alive	34 (87.2)	264 (86.8)	0.95
	Died	5 (12.8)	40 (13.2)	
Cause of death	Thyroid cancer	3 (60.0)	16 (40.0)	0.39
	Other causes	2 (40.0)	24 (60.0)	

Data are presented as number and percentage. Two-sided Chi-Square test was used. Statistical significance was set at *p*-value < 0.05. API: Asian or Pacific Islander, AI/AN: Am. Indian/Alaska Native, MTC: medullary thyroid cancer, M: million.

**Table 4:** Impact of the extension of surgery on survival in MTC patients.

Risk Factor	Overall Mortality			Thyroid Cancer-Specific Mortality		
	HR	95%CI	*p*-Value	HR	95%CI	*p*-Value
Age: ≥55 vs. <55 y	3.03	(1.66–5.52)	<0.001	2.63	(1.04–6.68)	0.042
Sex: male vs. female	1.35	(0.72–2.54)	0.35	1.26	(0.48–3.33)	0.64
Income: ≥USD 75 k vs. <USD 75 k	0.59	(0.30–1.16)	0.13	0.67	(0.25–1.83)	0.44
Residency: rural vs. urban	1.06	(0.43–2.59)	0.90	1.07	(0.23–4.99)	0.93
T stage: T1b vs. T1a	1.32	(0.70–2.48)	0.39	0.89	(0.34–2.30)	0.81
Nodal infiltration: N1 vs. N0	3.80	(2.03–7.10)	<0.001	7.76	(2.66–22.61)	<0.001
Total vs. subtotal/lobectomy	0.77	(0.29–2.03)	0.60	0.44	(0.11–1.68)	0.23

Cox regression analysis was performed and hazards ratio (HR) and 95% confidence interval (CI) were reported.

**Table 5:** Impact of radiation and systemic therapy on survival.

Treatment	Model	Overall Mortality				Thyroid Cancer-Specific Mortality			
		HR	LL	UL	*p*-Value	HR	LL	UL	*p*-Value
Radioactive ablation	Univariate	1.49	0.59	3.81	0.4	1.61	0.38	6.82	0.52
	Multivariate	1.33	0.50	3.51	0.57	1.63	0.36	7.42	0.53
Beam radiation	Univariate	4.42	1.57	12.48	0.005	3.45	1.02	14.64	0.049
	Multivariate	1.51	0.48	4.81	0.48	3.21	0.58	17.88	0.18
Systemic therapy	Univariate	0.01	1.00	0.94	0.94	0.30	0.04	2.32	0.25
	Multivariate	0.77	0.09	6.46	0.81	1.08	0.11	10.52	0.95

Multivariate Cox regression analysis was performed adjusting for age, sex, race, income, residency, T stage, and N stage. Hazards ratio (HR) and 95% confidence interval are reported.

**Table 6:** Survival times according to adjuvant therapy.

Group	Overall Survival Time				Thyroid Cancer-Specific Survival Time			
	Event/Total	Mean	95%CI	*p*-Value	Event/Total	Mean	95%CI	*p*-Value
Neck dissection								
Negative	53/352	199.6	189.9–209.3	0.07	25/324	216.7	208.2–225.1	0.12
Positive	7/46	218.4	204.2–232.7		2/42	231.5	222.8–240.2	
Radioactive iodine								
Negative	55/384	203.7	194.5–212.0	0.49	25/354	219.8	212.5–227.1	0.51
Positive	5/14	197.6	162.4–232.7		2/11	203.5	159.7–247.3	
Beam radiation								
Negative	56/386	204.7	196.3–213.1	0.030	25/355	220.6	213.5–227.6	0.047
Positive	4/12	144.2	93.7–194.7		2/10	163.0	104.5–221.5	
Systemic therapy								
Negative	59/286	201.4	192.4–210.3	0.15	26/253	217.7	209.6–225.6	0.22
Positive	1/112	144.5	141.5–147.4		1/112	144.5	141.5–147.4	

Log-rank test was used to compare between cohorts. Time in months.

**Table 7:** Comparison between total thyroidectomy and less aggressive surgery.

Characteristics	Levels	Early Treatment (N = 278)	Late Treatment (N = 74)	*p*-Value
Demographics				
Age (years)	Pediatric	28 (10.1)	0 (0.0)	0.001
	Adults	250 (89.9)	74 (100)	
	<55 years	179 (64.4)	33 (44.6)	0.003
	≥55 years	99 (35.6)	41 (55.4)	
Gender	Female	183 (65.8)	52 (70.3)	0.49
	Male	95 (34.2)	22 (29.7)	
Race	White	240 (86.3)	66 (89.2)	0.78
	Black	15 (5.4)	3 (4.1)	
	API	20 (7.2)	5 (6.8)	
	AI/AN	3 (1.1)	0 (0.0)	
Ethnicity	Not Hispanic/Latino	245 (88.1)	63 (85.1)	0.55
	Hispanic/Latino	33 (11.9)	11 (14.9)	
Marital status	Married	158 (61.7)	37 (54.4)	0.64
	Domestic partner	4 (1.6)	1 (1.5)	
	Separated	3 (1.2)	1 (1.5)	
	Divorced	13 (5.1)	5 (7.4)	
	Widowed	13 (5.1)	7 (10.3)	
	Single	65 (25.4)	17 (25.0)	
Metropolitan	Metropolitan >1 M pop	153 (55.0)	41 (55.4)	0.99
	Metropolitan >250 K^−1^ M	73 (26.3)	20 (27.0)	
	Metropolitan of <250 K	19 (6.8)	5 (6.8)	
	Non-Metropolitan adj to Metropolitan	20 (7.2)	5 (6.8)	
	Non-Metropolitan not adj to Metropolitan	13 (4.7)	3 (4.1)	
Household annual income	<USD 75,000	182 (65.5)	49 (66.2)	0.90
	≥USD 75,000	96 (34.5)	25 (33.8)	
Pathological data				
T staging	T1a	149 (53.6)	34 (45.9)	0.29
	T1b	129 (46.4)	40 (54.1)	
N staging	N0	196 (70.5)	43 (58.1)	0.05
	N1	82 (29.5)	31 (41.9)	
Management				
Cancer-specific surgery	Lobectomy/subtotal thyroidectomy	36 (13.5)	2 (2.8)	0.010
	Total thyroidectomy	231 (86.5)	70 (97.2)	
Surgery at other sites	Regional sites	47 (16.9)	6 (8.1)	0.07
	Regional lymph nodes	41 (14.7)	4 (5.4)	0.032
	Distant sites	17 (6.1)	4 (5.4)	0.82
	Distant lymph nodes	14 (5.0)	4 (5.4)	0.89
Radiation therapy	Negative	256 (92.1)	72 (97.3)	0.29
	Beam radiation	10 (3.6)	1 (1.4)	
	Radioactive ablation	12 (4.3)	1 (1.4)	
Radiation therapy with surgery	Positive	21 (7.6)	2 (2.7)	0.19
Systemic therapy with surgery	Positive	73 (26.3)	38 (51.4)	<0.001

Data are presented as number and percentage. Two-sided Chi-Square test was used. Statistical significance was set at *p*-value < 0.05. API: Asian or Pacific Islander, AI/AN: Am. Indian/Alaska Native, MTC: medullary thyroid cancer, M: million.

**Table 8:** Comparison between deceased and survived MTC patients with T1N0M0 stage.

Characteristics	Levels	Survived (N = 249)	Died (N = 31)	*p*-Value
Demographics				
Age (years)	<55 years	153 (61.4)	10 (32.3)	0.003
	≥55 years	96 (38.6)	21 (67.7)	
Gender	Female	171 (68.7)	19 (61.3)	0.42
	Male	78 (31.3)	12 (38.7)	
Race	White	212 (85.1)	25 (80.6)	0.49
	Black	19 (7.6)	2 (6.5)	
	API	15 (6.0)	4 (12.9)	
	AI/AN	3 (1.2)	0 (0.0)	
Ethnicit	Not Hispanic/Latino	215 (86.3)	29 (93.5)	0.39
	Hispanic/Latino	34 (13.7)	2 (6.5)	
Residency	Urban	217 (87.1)	27 (87.1)	0.99
	Rural	32 (12.9)	4 (12.9)	
Household annual income	<USD 75,000	157 (63.1)	24 (77.4)	0.16
	≥USD 75,000	92 (36.9)	7 (22.6)	
Pathological data				
T staging	T1a	162 (65.1)	20 (64.5)	0.95
	T1b	87 (34.9)	11 (35.5)	
Management				
Cancer-specific surgery	Negative	24 (9.6)	11 (35.5)	<0.001
	Positive	225 (90.4)	20 (64.5)	
Extent of surgery	Lobectomy/subtotal thyroidectomy	31 (14.4)	4 (21.1)	0.49
	Total thyroidectomy	184 (85.6)	15 (78.9)	
Surgery at other sites	Regional sites	33 (13.3)	2 (6.5)	0.39
	Regional lymph nodes	29 (11.6)	2 (6.5)	0.55
	Distant sites	7 (2.8)	0 (0.0)	0.34
	Distant lymph nodes	6 (2.4)	2 (6.5)	0.22
Radiation therapy	Negative	238 (95.6)	26 (83.9)	0.019
	Beam radiation	4 (1.6)	1 (3.2)	
	Radioactive ablation	7 (2.8)	4 (12.9)	
Radiation therapy with surgery	Positive	10 (4.0)	5 (16.1)	0.016
Systemic therapy with surgery	Positive	67 (26.9)	1 (3.2)	0.002
Time to treatment	Early treatment	180 (81.8)	16 (84.2)	0.79
	Delayed treatment	40 (18.2)	3 (15.8)	
Disease outcomes				
Recurrence	Positive recurrence	1 (0.4)	0 (0.0)	0.72
Second primary malignancy	Any cancer	33 (13.3)	4 (12.9)	0.95
	Thyroid cancer	11 (4.4)	0 (0.0)	0.62

Data are presented as number and percentage. Two-sided Chi-Square test was used. Statistical significance was set at *p*-value < 0.05. API: Asian or Pacific Islander, AI/AN: Am. Indian/Alaska Native, MTC: medullary thyroid cancer, M: million.

**Table 9:** Independent risk factors for survival in early-stage MTC without nodal metastasis (T1N0).

Predictor Risk Factors	Overall Mortality				Thyroid Cancer-Specific Mortality			
	HR	LL	UL	*p*-Value	HR	LL	UL	*p*-Value
Age (years)	1.073	1.03	1.118	<0.001	1.029	0.969	1.093	0.347
Gender: male vs. female	0.36	0.098	1.323	0.124	0.374	0.038	3.646	0.397
Rural vs. urban residency	1.147	0.272	4.829	0.852	0.723	0.065	8.045	0.792
High vs. low income	0.302	0.083	1.098	0.069	0.969	0.154	6.104	0.973
T stage: T1b vs. T1a	1.31	0.465	3.691	0.61	0.253	0.028	2.249	0.218
Total thyroidectomy vs. lobectomy/subtotal	0.805	0.223	2.898	0.74	0.222	0.036	1.359	0.103
From Dx to Treatment (months)	0.824	0.454	1.496	0.524	1.081	0.411	2.842	0.874
Age (years)	1.073	1.03	1.118	<.001	1.029	0.969	1.093	0.347
Gender: male vs. female	0.36	0.098	1.323	0.124	0.374	0.038	3.646	0.397
Rural vs. urban residency	1.147	0.272	4.829	0.852	0.723	0.065	8.045	0.792

Cox regression test was performed, and hazards ratio (HR) and 95% confidence intervals are reported.

## Data Availability

Publicly available datasets were analyzed in this study. These data can be found here: https://seer.cancer.gov (accessed on 29 September 2023).
